# Alizarin Red S-Functionalized Polyurethane Coatings as Passive Colorimetric Interfaces for Visual Heavy-Metal Screening in Simulated Seawater

**DOI:** 10.3390/polym18182294

**Published:** 2026-09-19

**Authors:** Silvia Sfameni, Mariam Hadhri, Giulia Rando, Agnese D’Agostino, Raphael Palucci Rosa, Giuseppe Rosace, Valentina Trovato, Maria Rosaria Plutino

**Affiliations:** 1 Institute for the Study of Nanostructured Materials—National Research Council (ISMN–CNR), URT Messina, c/o Department of ChiBioFarAm, University of Messina, Viale F. Stagno d’Alcontres 31, 98166 Messina, Italy; silvia.sfameni@cnr.it (S.S.); giulia.rando@cnr.it (G.R.); 2 Department of Engineering and Applied Sciences, University of Bergamo, Viale Marconi 5, 24044 Dalmine, Italy; mariam.hadhri@unibg.it (M.H.); agnese.dagostino@unibg.it (A.D.); raphael.rosa@unibg.it (R.P.R.); giuseppe.rosace@unibg.it (G.R.); 3 Local CSGI (Inter-University Center for Colloid and Surface Science) Research Unit, 24044 Dalmine, Italy

**Keywords:** Alizarin Red S, polyurethane coating, heavy-metal ions, simulated seawater, colorimetric screening, supported optical interface

## Abstract

This study investigates Alizarin Red S (ARS)-functionalized polyurethane (PU) coatings as passive colorimetric interfaces for preliminary visual screening of selected heavy-metal ions in simulated seawater. ARS was incorporated into a hydrophilic PU matrix and deposited on glass substrates. UV–visible spectroscopy and ATR-FTIR were used to analyze the optical and vibrational features of the PU–ARS system, while DLS provided comparative information on the colloidal behavior of the liquid precursor formulations before film formation. After 48 h exposure to simulated seawater (ASTM D1141-98, pH 8.2) containing Pb^2+^, Cd^2+^, Ni^2+^, or Hg^2+^ at 5 × 10^−4^ M, the coatings developed ion-dependent color changes quantified in the CIE L*a*b* color space. Under these single-ion conditions, the total color difference followed the order Pb^2+^ > Cd^2+^ > Ni^2+^ > Hg^2+^, with approximate ΔE values of 27, 19, 16, and 13, respectively. Saline pre-immersion experiments up to 72 h, followed by 48 h exposure to metal-ion solutions, indicated that the coatings retained macroscopic readability and optical responsiveness under the tested conditions. The results support the use of PU–ARS coatings as proof-of-concept passive interfaces for visual heavy-metal screening in marine-like media. Further studies including calibration, detection limits, selectivity, interference, leaching assessment, and validation in real seawater will be required before analytical implementation.

## 1. Introduction

Heavy-metal contamination is a persistent and analytically demanding problem in aquatic environments. Unlike organic pollutants, metals are not mineralised: once released, they partition among dissolved, colloidal, and particulate phases, enter biological systems, and accumulate in aquatic organisms and food webs [1,2]. Therefore, environmental risk cannot be assessed solely on the basis of total metal concentration, as it depends also on chemical speciation, residence time, exposure pathway, and bioavailability [3,4].

In marine and coastal waters, the analytical assessment of heavy metal contamination is further complicated by the chemical complexity inherent in the matrix. High ionic strength, abundant competing cations (such as Na^+^, Mg^2+^, Ca^2+^, K^+^), chloride-rich chemistry, variable pH levels and dissolved organic matter all affect metal speciation, complex formation, and the apparent response of sensors [5,6]. The concentration of free hydrated metal ions may differ substantially from the nominal added concentration because complexation with chloride, carbonate, hydroxide, and dissolved organic ligands redistributes metals among different inorganic and organic species. In the case of a colorimetric coating, this means that the observed response mirrors interactions between the film and the metal species available under the selected matrix conditions, rather than the total metal concentration [5]. Reference analytical techniques, such as inductively coupled plasma mass spectrometry, atomic absorption spectroscopy, and electrochemical stripping methods, are still essential for precise trace-metal measurement. However, these methods generally require controlled laboratory environments, trained personnel, and matrix-specific correction or preconcentration procedures. Electrochemical platforms have achieved significant progress in enabling on-site heavy metal detection and provide high sensitivity. Nevertheless, their effectiveness in complex media continues to depend on electrode architecture, fouling resistance, and calibration strategy [7]. These constraints permit the use of supplementary low-cost optical materials designed for initial sample screening, particularly when the aim is to identify potentially contaminated sites or samples that necessitate subsequent laboratory confirmation. Within this framework, polymer-based coatings provide a practical route to immobilize chromogenic probes at solid–liquid interfaces, while preserving processability, mechanical handling, and compatibility with aqueous environments. Previous studies on functional polymeric and hybrid coatings have shown that appropriately designed organic–inorganic or polymer-based matrices can provide durable interfaces for environmental, textile, and surface-protection applications [8,9,10,11].

Additionally, colorimetric sensing offers significant advantages for preliminary monitoring because the signal can be read by direct visual inspection, reflectance measurements, or digital image analysis [12,13,14,15,16,17,18]. However, apparent simplicity can be misleading: in the context of heavy-metal sensing, the response is influenced not only by receptor–analyte interactions but also by factors such as immobilization chemistry, matrix permeability, dye retention, optical contrast, and resistance to interfering ions [12,13,14]. Polymeric films provide an attractive platform by confining molecular probes in a defined solid phase that provides a mechanically robust interface with the aqueous medium. For dye-based probes, immobilization can reduce probe migration and improve handling while still permitting analyte diffusion if the host matrix is sufficiently permeable.

Alizarin Red S (ARS), sodium 3,4-dihydroxy-9,10-dioxoanthracene-2-sulfonate, is a water-soluble anthraquinone dye with well-characterized optical and coordination properties [19]. Its chromogenic behavior arises from the conjugated anthraquinone scaffold and the presence of adjacent hydroxyl and carbonyl groups, along with a sulfonate substituent. These features collectively facilitate pH-dependent optical transitions and provide coordination sites for metal-ion interactions. At a mildly alkaline pH, ARS undergoes partial deprotonation of phenolic sites, and this acid–base behavior is closely associated with alterations in the electronic structure of the anthraquinone chromophore. Metal coordination or ion pairing may induce color changes not only through direct binding but also through modulation of the local protonation state and microenvironment of the dye [20,21]. At pH 8.2, the coating should be regarded as a hydrated and ion-containing environment rather than as a static assembly of neutral molecules. Reported acid–base data for ARS give pKa values of approximately 5.82 and 10.78 for the two phenolic deprotonation steps [22]. At pH 8.2, the mono-deprotonated phenolic form is therefore expected to predominate in aqueous solution, in addition to the permanently ionized sulfonate group. Immobilization within the hydrated PU phase may modify this local acid–base environment, so the solution-phase speciation is used here only as a reference for discussing the coating. At the same time, the anionic hydrophilic polyurethane contains ionized groups that stabilize the waterborne dispersion and contribute to water uptake, ion exchange, and local electrostatic interactions in the film. The observed color response is therefore governed not only by possible ARS–metal coordination but also by ARS protonation state, hydration, ion pairing, and the polar microenvironment provided by the PU matrix.

ARS has previously been employed in solution-phase and hybrid sensing systems, including alizarin-phenylboronic acid ensembles and ARS-functionalized graphene quantum dots for pH- and cation-responsive colorimetric assays [21,23,24]. These examples highlight the versatility of ARS as a chromogenic receptor, but they leave open a practical issue that becomes central for immersed sensing materials: the dye must remain optically active while confined within a mechanically stable solid phase. In this context, the key question is whether the response of ARS to metal ions in solution remains intact after immobilization within a coating exposed to saline media. Waterborne polyurethanes are extensively utilized as film-forming agents due to their ability to be tailored to enhance flexibility, adhesion, hydrophilicity, and chemical resistance [25]. In a sensing coating, a hydrophilic polyurethane phase allows aqueous metal ions to diffuse towards the embedded chromophore, whilst the polymer network retains the dye at the solid–liquid interface and ensures mechanical stability. This combination is directly pertinent to immobilized colorimetric interfaces, where performance is influenced not solely by receptor–analyte binding but also by factors such as film integrity, swelling, optical contrast, probe retention, and matrix resistance.

The present study investigates the use of an anionic hydrophilic polyether-polyurethane resin as a host matrix for ARS, with the aim of maintaining its chromophoric response and producing a visually distinguishable colorimetric coating following exposure to conditions analogous to seawater. ARS was incorporated into the PU matrix and then deposited onto glass substrates; consequently, the coatings were characterized before and after exposure to individual metal ions of Pb^2+^, Cd^2+^, Ni^2+^, and Hg^2+^ in simulated seawater. UV–visible spectroscopy, dynamic light scattering (DLS), attenuated total reflection Fourier transform infrared spectroscopy (ATR-FTIR), Scanning Electron Microscopy coupled with Energy-Dispersive X-ray Spectroscopy (SEM-EDS), and reflectance colorimetry in the CIE L*a*b* space were combined to investigate dye–polymer interactions, coating morphology, surface elemental composition, and ion-driven chromatic changes. This work is presented as an initial feasibility study of a PU-supported ARS coating under controlled single-ion exposure conditions; analytical figures of merit, mixed-ion selectivity, reversibility, leaching behavior, and real-sample validation remain outside the scope of the present study.

## 2. Materials and Methods

### 2.1. Materials and Coating Preparation

#### 2.1.1. Materials

All reagents and solvents were used as received unless otherwise specified. The anionic hydrophilic polyether-polyurethane resin RESILFIX MPC 50 was supplied by F.T.R. S.r.l. (Albano Sant’Alessandro, Italy). Absolute ethanol was purchased from Merck Chemicals (Milan, Italy). Nickel (II) nitrate hexahydrate (Ni(NO_3_)_2_·6H_2_O, ≥98%) was obtained from Alfa Aesar (Milan, Italy), mercury(II) nitrate monohydrate (Hg(NO_3_)_2_·H_2_O, 98.5%) and Alizarin Red S (ARS, C_14_H_7_NaO_7_S) from Sigma-Aldrich (Milan, Italy), cadmium(II) acetate dihydrate (Cd(CH_3_COO)_2_·2H_2_O, 99%) from Carlo Erba (Cornaredo, Italy), and lead(II) nitrate (Pb(NO_3_)_2_, 99%) from Acros Organics (Geel, Belgium). Simulated seawater was prepared according to ASTM D1141-98 [26] and adjusted to pH 8.2 ± 0.1 before use.

#### 2.1.2. Coating Preparation

A mass ratio of 2:1:1 (polyurethane dispersion:distilled water:ethanol, *w*/*w*/*w*) was adopted to ensure uniform film deposition. Polyurethane dispersion (1.0 g) was mixed with 0.5 g of distilled water and stirred for 10 min to obtain a homogeneous aqueous phase. Ethanol (0.5 g) was then added dropwise to reduce surface tension and enhance substrate wettability [27]. ARS was subsequently incorporated at a concentration of 7.30 × 10^−3^ M, and the mixture was stirred continuously at room temperature until a macroscopically homogeneous dispersion was obtained. The final formulation was applied to one side of microscope glass slides (Brand GmbH + CO KG, Wertheim, Germany) by brush coating and allowed to dry at room temperature for 15 days. This drying period was used to obtain macroscopically continuous films before characterization and immersion testing.

#### 2.1.3. Preparation of Metal-Enriched Simulated Seawater and Immersion Protocol

Stock solutions of Pb^2+^, Ni^2+^, Cd^2+^, and Hg^2+^ were prepared from the respective salts described in Section 2.1.1 and diluted with simulated seawater to obtain a final concentration of 5 × 10^−4^ M for each metal ion. This concentration was selected as a deliberate stress-test concentration to produce clearly measurable and visually distinguishable colorimetric responses in a high-ionic-strength matrix. It exceeds established environmental limits and was therefore not intended to represent environmentally relevant concentrations or analytical detection limits. PU–ARS-coated glass slides were immersed in the metal-enriched solutions at room temperature for 48 h. The coated area was kept constant for all samples, but the dry coating mass and the amount of ARS deposited per slide were not independently determined. This prevents calculation of the exact metal/ARS molar ratio and is therefore recognized as a limitation of the present proof-of-concept study.

A PU–ARS-coated slide immersed in metal-free simulated seawater under identical conditions served as the reference. In parallel, the effect of saline pre-immersion on coating integrity and retention of the chromogenic response was evaluated by pre-immersing independently prepared PU–ARS-coated slides in metal-free simulated seawater for 8, 16, 24, 48, and 72 h. After each pre-immersion time, the coatings were transferred into the selected metal-ion solutions and exposed for an additional 48 h. Reflectance colorimetry was then performed after metal-ion exposure to assess whether prior saline immersion affected the subsequent optical response. Following exposure, the coated samples were carefully removed, drained without mechanical wiping, and analyzed by photography and reflectance colorimetry. All colorimetric measurements were performed on at least three independently prepared coated slides for each condition, and the results are reported as mean values with standard deviations.

### 2.2. UV–Visible Characterization of Liquid Formulations and Solid Coatings

UV–visible absorption spectra were recorded using an Evolution OnePlus spectrophotometer (Thermo Scientific, Waltham, MA, USA) and Insight™ Pro (version 3.0.0.109) software over the wavelength range 350–800 nm, with a spectral resolution of 0.5 nm, using quartz cuvettes with 1 cm optical path length.

**Figure 1:**
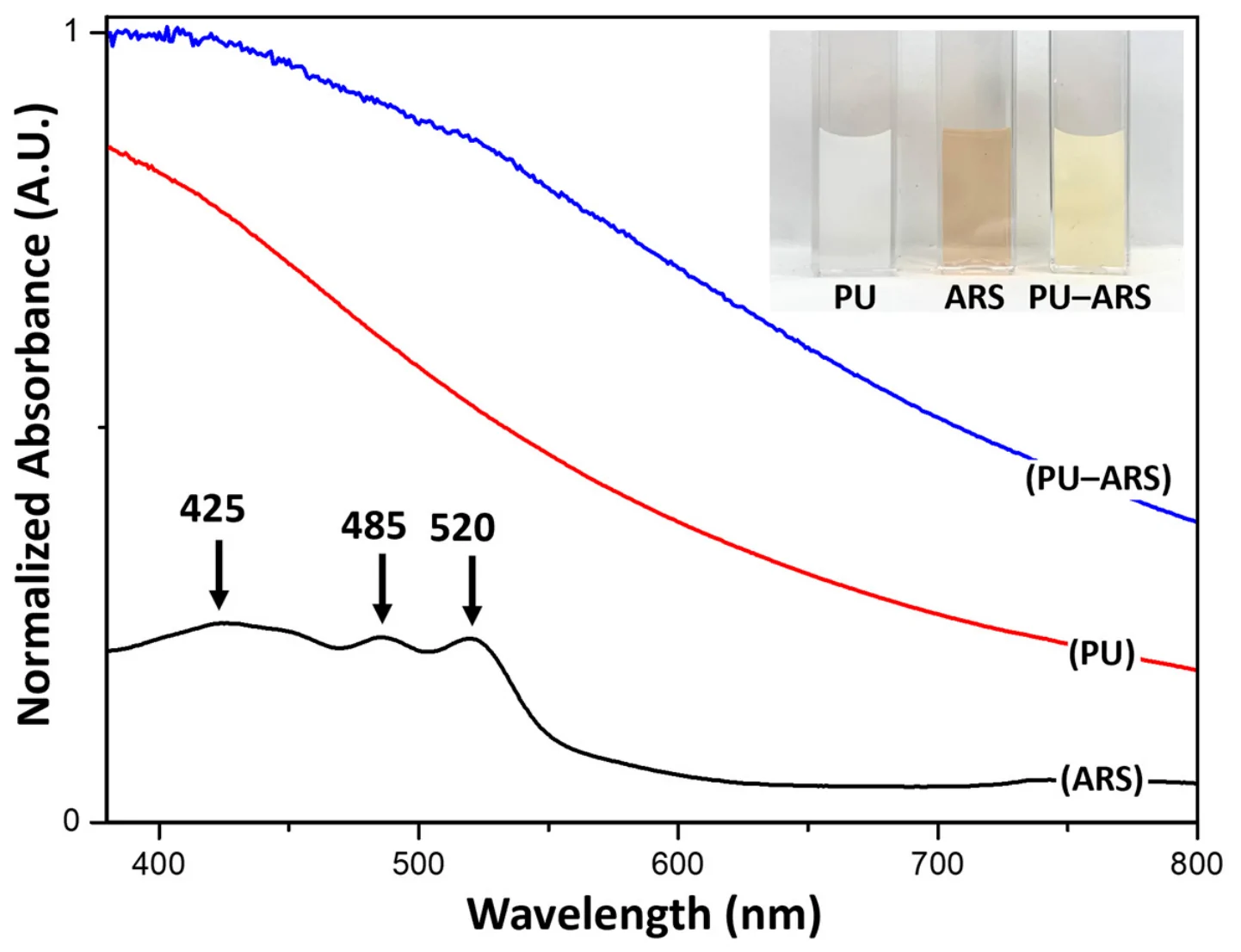
Normalized UV–visible absorbance spectra of the aqueous Alizarin Red S (ARS) solution, pristine polyurethane (PU), and PU–ARS hybrid precursor formulation, along with photographs of the corresponding liquid systems. Spectra were normalized to facilitate comparison of band positions and spectral profiles.

For the liquid-phase measurements shown in Figure 1, the ARS solution, pristine PU dispersion, and PU–ARS precursor formulation were diluted 1:100 with deionized water and analyzed in quartz cuvettes with a 1 cm optical path length. The final ARS concentration in the diluted ARS solution was approximately 7.3 × 10^−5^ M. The PU and ARS reference samples reproduced the corresponding component concentrations and solvent compositions of the coating formulation and underwent the same dilution procedure. For the solid-state measurements, PU–ARS coatings deposited on glass were analyzed before and after metal exposure. A dried ARS reference was prepared separately by depositing an ARS solution in a 1:1 (*w*/*w*) water/ethanol mixture on glass and allowing the solvent to evaporate at room temperature. PU–ARS coating spectra were recorded against a blank PU-coated glass slide, whereas an uncoated glass slide was used as the background for the dried ARS reference. Liquid- and solid-state spectra were normalized and compared separately.

Particle size distributions of the PU-blank dispersion, ARS solution, and PU–ARS formulation were evaluated by dynamic light scattering (DLS) using a Zetasizer Pro (Malvern Instruments Ltd., Worcester, UK). For DLS measurements, 2 mL of each sample was diluted tenfold (1:10, *v*/*v*) with distilled water, transferred into polystyrene cuvettes (10 × 10 × 45 mm, Sarstedt, Nürnbrecht, Germany), and analyzed at 25 °C. DLS measurements provide intensity-weighted hydrodynamic diameters and are not directly applicable to molecular-scale species; therefore, the obtained values were interpreted as comparative indicators of colloidal dispersion behavior rather than absolute particle dimensions.

### 2.3. Characterization of Coatings

ATR-FTIR spectra were recorded on a Thermo Avatar 370 spectrometer (Thermo Nicolet Corp., Madison, WI, USA) equipped with a diamond crystal ATR accessory. Spectra were collected over the 4000–650 cm^−1^ spectral range, averaging 32 scans per spectrum at a resolution of 4 cm^−1^, and are reported in transmittance units. The analyzed samples included ARS powder, PU-coated glass substrates, and PU–ARS-coated glass substrates, both before and after metal-ion exposure.

The colorimetric response of PU–ARS coatings was assessed using a Datacolor Spectro 700 reflectance spectrophotometer (Datacolor Italia srl, Giussano, Italy). The CIE L*, a*, and b* coordinates were recorded for each condition, and the total color difference, ΔE, was calculated relative to the metal-free seawater reference according to:

(1) $$\Delta E=\sqrt{{\left(L^{*}-L_{0}^{*}\right)}^{2}+{\left(a^{*}-a_{0}^{*}\right)}^{2}+{\left(b^{*}-b_{0}^{*}\right)}^{2}}$$

where L_0_*, a_0_*, and b_0_* are the CIE coordinates of the PU–ARS coating immersed in metal-free simulated seawater, and L*, a*, and b* correspond to the coordinates measured after metal-ion exposure. The instrument was calibrated before each measurement session using the manufacturer’s white and black standards. For each coated slide, three spatially separated points were measured, and the reported value corresponds to the average of these readings. According to established color-difference criteria [28,29], ΔE < 1 indicate imperceptible differences, values between 1 and 3 are generally detectable by trained observers, whereas values above 3 represent clearly visible color changes. Surface morphology was examined by scanning electron microscopy (SEM) using a Phenom ProX G6 Desktop SEM (Thermo Fisher Scientific, Segrate, Italy). Elemental composition of the analyzed surface areas was assessed using the integrated energy-dispersive X-ray spectroscopy (EDS) detector. SEM-EDS analyses were performed at an accelerating voltage of 15 kV. Prior to analysis, specimens were mounted on aluminum stubs with conductive adhesive tape and coated with a 5 nm gold layer using a Luxor Au sputter coater (APTCO, Berlin, Germany) in a nitrogen (N_2_) atmosphere.

## 3. Results

### 3.1. Characterization of ARS, PU-Blank, and PU–ARS

Before assessing the metal-induced response, the PU-blank and PU–ARS systems were first characterized to evaluate the effect of ARS incorporation on the optical, colloidal, and vibrational properties of the precursor formulation and the resulting coating. This preliminary step is essential because the colorimetric behavior of a solid film is not governed solely by the intrinsic metal affinity of the embedded dye. Dye dispersion state, dye–polymer interactions, local accessibility of the chromophore, and film morphology can all modulate the observed response. UV–visible spectroscopy, DLS, and ATR-FTIR were therefore used to define the baseline features of the PU–ARS system prior to metal-ion exposure.

#### 3.1.1. Optical Response and Colloidal Characterization of ARS, PU and PU–ARS Formulations

The UV–visible spectra of Alizarin Red S (ARS), the pristine polyurethane (PU) dispersion, and the PU–ARS hybrid formulation are reported in Figure 1.

The ARS spectrum shows three visible absorption features at approximately 425, 485 and 520 nm, reflecting the pH- and environment-dependent optical response of the hydroxyanthraquinone chromophore. Comparable ARS absorption bands have been reported around 420 and 517 nm in pH-dependent studies, whereas an absorption maximum near 428 nm has been observed for ARS in aqueous solution, with shifts occurring as a function of the polarity and dielectric properties of the surrounding medium [22,30].

The weak feature observed at approximately 485 nm may reflect the complex pH-dependent spectral response of ARS, although a definitive assignment is not possible from the present measurements. Therefore, the observed features can be conservatively assigned to electronic transitions of the anthraquinone-based chromophore, predominantly involving π–π* excitations, while lower-intensity n–π* contributions cannot be excluded. A more specific assignment of the individual features would require analysis of ARS speciation and dedicated spectroscopic or computational investigations [31].

The pristine PU dispersion exhibits a broad, monotonically decreasing, and essentially featureless signal over the visible region. This profile should be interpreted primarily as optical extinction, with a significant contribution from light scattering, rather than as a well-defined electronic absorption of the polymer. Following ARS incorporation into the PU dispersion, the PU–ARS spectrum is dominated by the broad extinction background of the polyurethane dispersion, which prevents the unambiguous resolution of the characteristic ARS absorption features. Consequently, the present spectra do not allow for the reliable assessment of possible changes in the position or shape of the ARS absorption bands. Such an analysis would require appropriate correction of the PU scattering background and, where justified, spectral subtraction or deconvolution. Nevertheless, the photograph in Figure 1 shows that the PU–ARS formulation exhibits a distinct visible coloration compared with the pristine PU dispersion. This qualitative observation is consistent with previous reports showing that ARS or alizarin-based chromophores can preserve their optical response after immobilization in hybrid or polymer-related matrices [32,33,34]. Overall, the UV-Vis spectrum of the PU–ARS formulation is governed primarily by the wavelength-dependent optical extinction of the polyurethane dispersion. Under these conditions, the contribution of ARS cannot be isolated unambiguously, and the spectra therefore do not provide sufficient evidence to distinguish among changes arising from the local environment, dye speciation, or aggregation [35].

**Figure 2:**
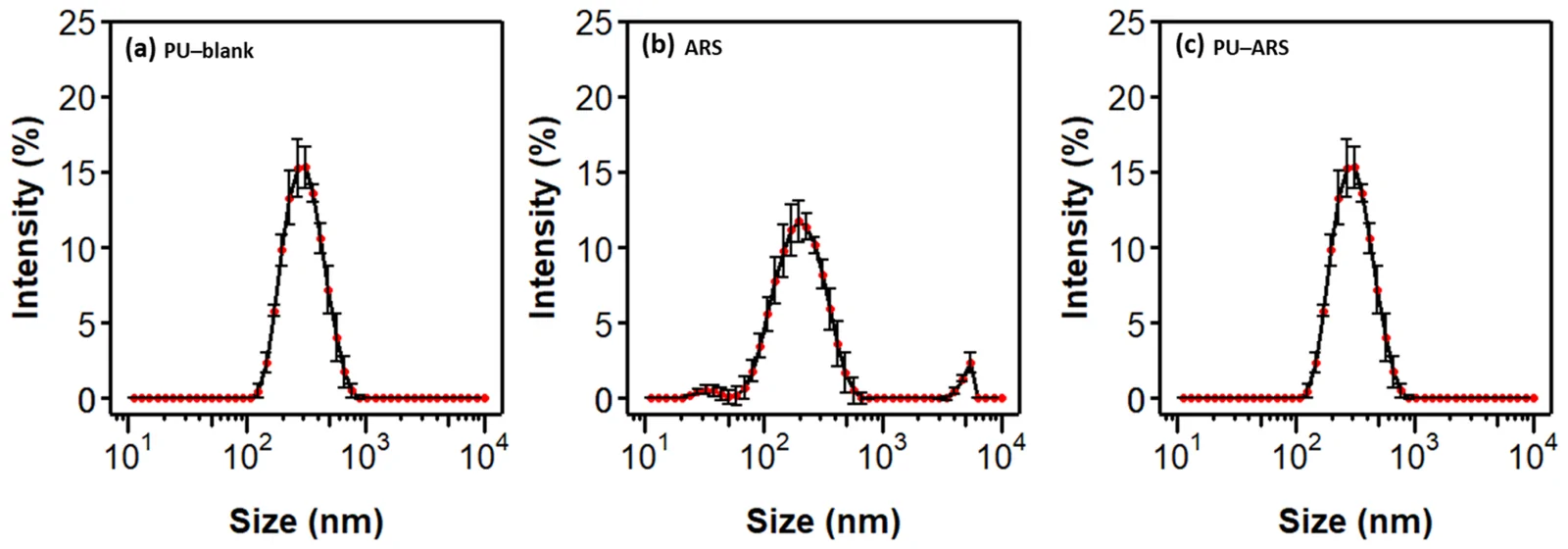
Hydrodynamic size distributions for PU–blank (**a**), ARS solution (**b**), and PU–ARS formulation (**c**).

DLS analysis was used to compare the colloidal properties of the PU-blank dispersion, the ARS solution, and the PU–ARS formulation (Figure 2). The PU-blank sample showed a primarily monomodal intensity-weighted size distribution, with the main hydrodynamic population centered at approximately 100 nm, consistent with a well-dispersed waterborne polyurethane dispersion. For the ARS solution, an apparent scattering population was detected, together with a minor contribution at larger hydrodynamic sizes. This signal is more appropriately attributed to dye-associated species or small aggregates than to isolated molecular ARS, because DLS is intrinsically poorly suited to molecular-scale solutes and responds preferentially to larger scattering objects [36]. After ARS incorporation, the PU–ARS formulation showed a shift in the main intensity-weighted population towards approximately 200 nm, accompanied by a broader size distribution. This behavior is compatible with dye-induced changes in the colloidal organization of the PU dispersion, possibly involving dye–polymer association and partial restructuring or aggregation of the dispersed PU domains, but should not be interpreted as evidence for a specific encapsulation geometry or a defined binding stoichiometry. DLS provides useful comparative colloidal information in this context but cannot resolve molecular-level dye–polymer interactions [36,37]. These measurements describe only the liquid precursor formulations and do not provide structural information on the dried coating.

#### 3.1.2. ATR-FTIR Characterization of Coatings

**Figure 3:**
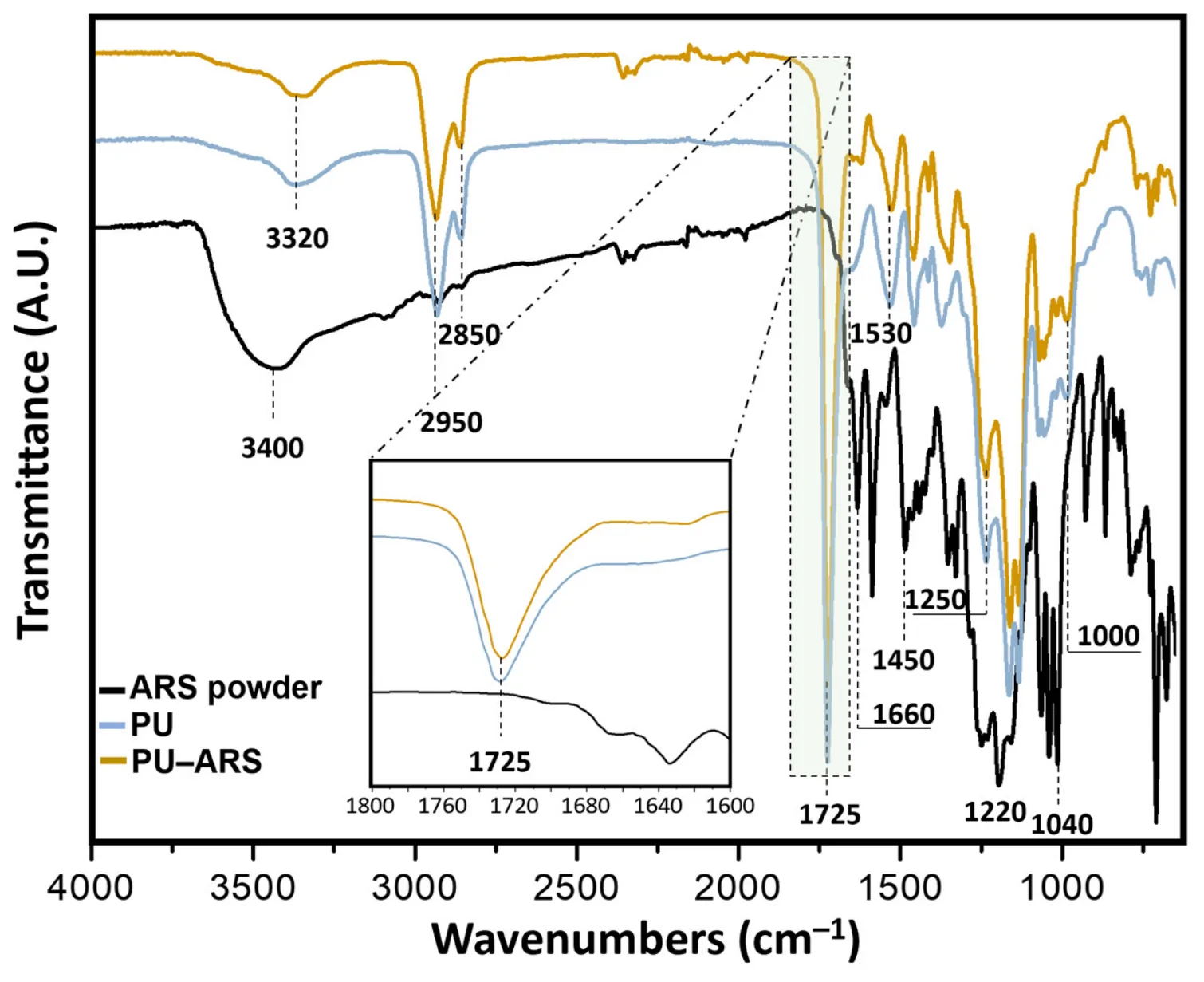
ATR-FTIR spectra of ARS powder, pristine PU coating on glass, and PU–ARS coating on glass. The inset shows an enlarged view of the 1800–1600 cm^−1^ carbonyl region.

ATR-FTIR spectra of ARS powder, pristine PU, and the PU–ARS coating are shown in Figure 3. At the investigated ARS loading, the PU–ARS spectrum is dominated by polyurethane bands, and overlap between the two components prevents unambiguous identification of ARS-specific dye–polymer interactions. In the ARS spectrum, the broad absorption in the 3600–3000 cm^−1^ region is assigned to hydrogen-bonded O–H stretching vibrations involving water molecules and phenolic groups. Bands around 1660, 1575, and 1040 cm^−1^ can be mainly attributed to anthraquinone C=O stretching, aromatic C=C stretching, and sulfonate-related vibrations, respectively, although coupling with phenolic C–O and ring modes cannot be excluded [38].

The PU spectrum shows features characteristic of segmented polyurethane: a broad N–H stretch at approximately 3320 cm^−1^, aliphatic C–H stretches between 2950 and 2850 cm^−1^, a urethane C=O band at 1725 cm^−1^, N–H bending near 1530 cm^−1^, and prominent C–O–C stretching in the 1100–1000 cm^−1^ region [39].

Within the PU–ARS coating spectrum, the regions corresponding to the carbonyl and O–H/N–H exhibit broadening and partial overlap of contributions associated with polyurethane and ARS. At the ARS loading used in the formulation, these overlapping bands do not allow for the reliable assignment of a resolved shift in the urethane carbonyl band from 1725 to approximately 1715 cm^−1^ [40]. Similarly, ARS-related contributions in the 1250–1000 cm^−1^ region overlap with polyurethane bands. Therefore, the spectra describe the combined vibrational profile of the PU–ARS coating and do not provide conclusive evidence of a specific dye–polymer interaction or hydrogen-bonding motif.

Changes in band shape within polyurethane spectra can reflect variations in hydrogen bonding and local polarity [41], but the present spectra do not allow these contributions to be separated from ARS band overlap or other matrix effects.

**Scheme 1:**
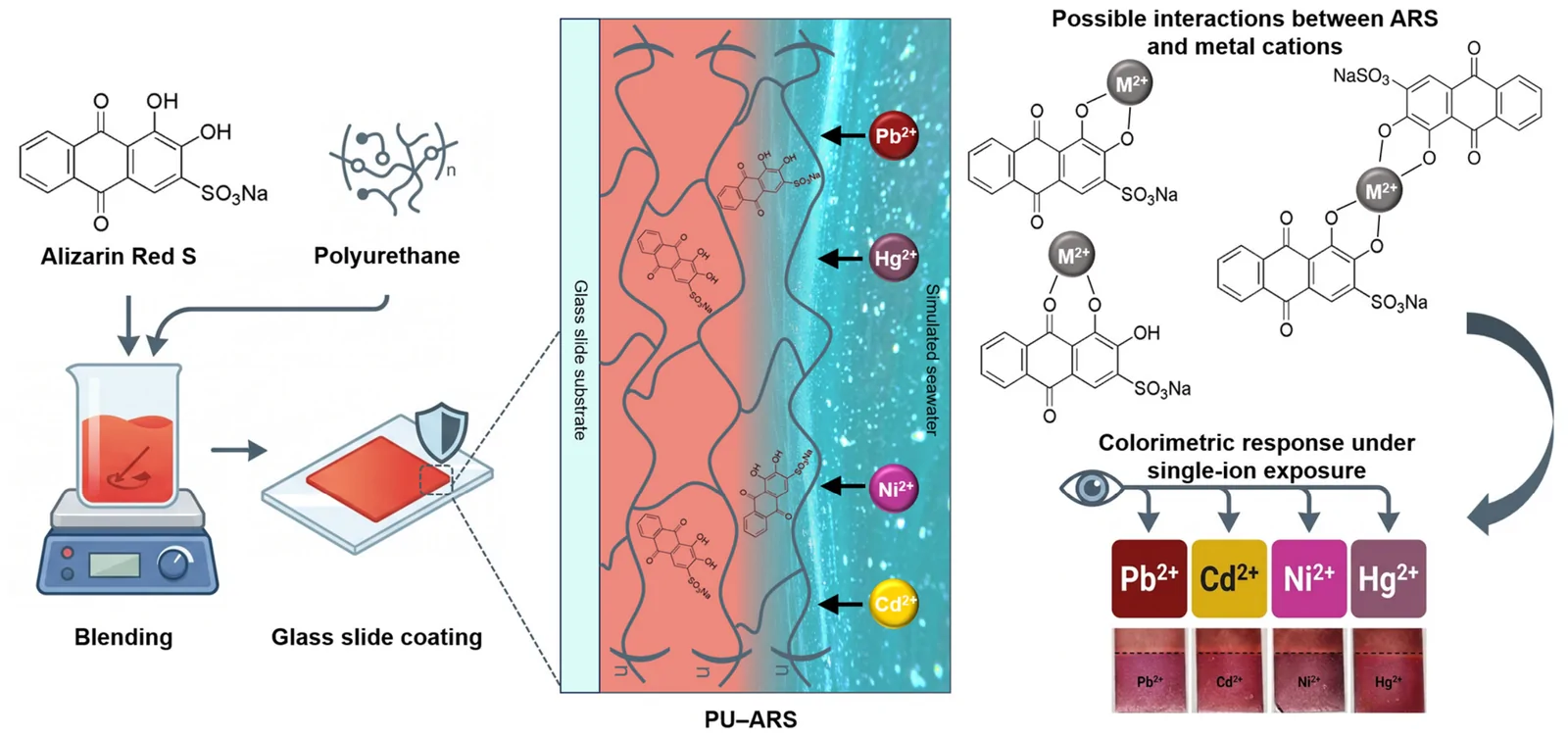
Proposed coating-level interaction model of ARS incorporated in the hydrophilic polyurethane matrix and possible metal-induced perturbations after exposure to divalent metal ions in simulated seawater.

Scheme 1 represents plausible interactions at the coating level, including hydrogen bonding, hydration, ion pairing, and perturbation of ARS hydroxyl, carbonyl and sulfonate groups. It is not intended as proof of a single coordination structure.

Overall, the FTIR data support the incorporation of ARS into the polyurethane film and show that its functional groups remain detectable in the solid state. At this ARS loading, ATR-FTIR was not used to assess dye retention after immersion.

### 3.2. Structural and Optical Response of PU–ARS Exposed to Heavy-Metal Ions

The spectroscopic response of PU–ARS coatings to exposure to divalent metal ions was investigated using ATR-FTIR and UV–visible transmission spectroscopy. The aim was to determine whether contact with Pb^2+^, Cd^2+^, Ni^2+^, or Hg^2+^ in simulated seawater produced detectable changes in the vibrational or optical signatures of the immobilized ARS chromophore. The following discussion focuses on coating-level spectral variations after single-ion exposure and deliberately avoids assigning definitive binding stoichiometries or coordination geometries, which would require dedicated solution-phase titration experiments and complementary structural methods.

**Figure 4:**
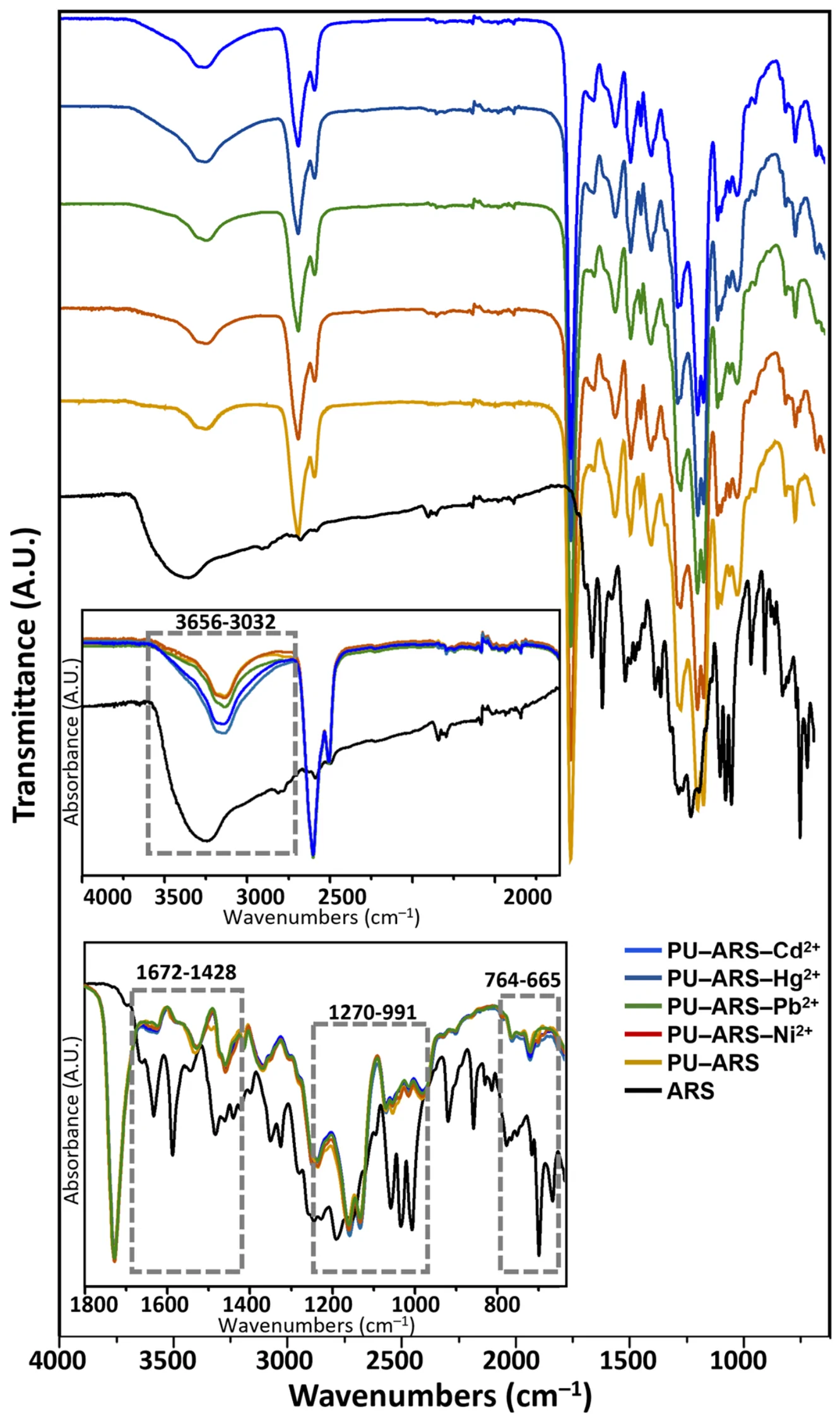
ATR-FTIR spectra of PU–ARS coatings before and after 48 h exposure to simulated seawater containing Cd^2+^, Hg^2+^, Pb^2+^, or Ni^2+^ at 5 × 10^−4^ M.

The ATR-FTIR spectra of PU–ARS coatings before and after exposure to simulated seawater containing Cd^2+^, Hg^2+^, Pb^2+^, and Ni^2+^ are presented in Figure 4, with the pristine PU–ARS coating used as the reference spectrum of the sensing layer before metal-ion exposure.

For the PU–ARS spectrum, the broad band in the 3650–3030 cm^−1^ region is assigned to overlapping hydrogen-bonded O–H and N–H stretching vibrations from ARS and urethane groups, respectively, while the bands at 2950–2850 cm^−1^ arise from aliphatic C–H stretching of the polyurethane soft segments. The carbonyl region is mainly dominated by urethane C=O stretching around 1725–1715 cm^−1^, with possible overlap from the quinonoid C=O stretching of ARS. The 1640–1450 cm^−1^ region includes ARS aromatic C=C vibrations and urethane N–H/C–N contributions, whereas the 1270–1000 cm^−1^ fingerprint region contains C–O–C, C–N/C–O, phenolic C–O and sulfonate S=O vibrations [38,39,41].

After exposure to metal-enriched simulated seawater, changes in band shape and relative intensity were observed in the O–H/N–H, carbonyl/aromatic, and fingerprint regions. These regions contain overlapping contributions from PU, ARS, water, and residual salts. Without an exposed PU-blank control or difference spectra, the changes cannot be assigned specifically to ARS–metal coordination and are therefore treated here as coating-level spectral variations associated with immersion in the metal-containing media.

**Figure 5:**
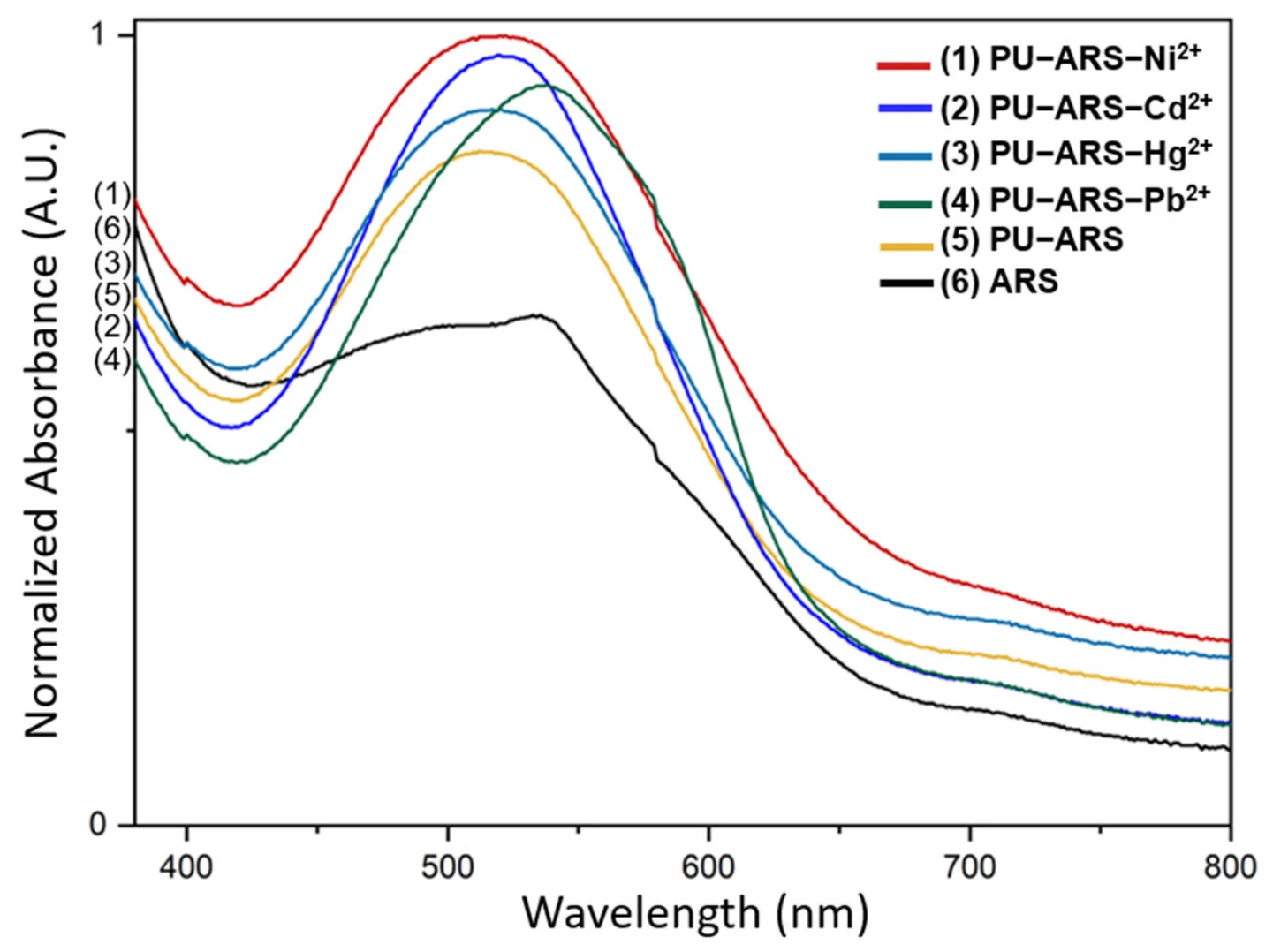
Normalized UV–visible absorbance spectra of PU–ARS coatings before and after exposure to simulated seawater containing Ni^2+^, Cd^2+^, Hg^2+^, or Pb^2+^ at 5 × 10^−4^ M, together with the ARS reference spectrum: (1) PU–ARS–Ni^2+^; (2) PU–ARS–Cd^2+^; (3) PU–ARS–Hg^2+^; (4) PU–ARS–Pb^2+^; (5) PU–ARS before metal exposure; and (6) ARS reference. Numerical identifiers are included in the plot legend to facilitate interpretation independently of color.

UV–visible spectroscopy of the PU–ARS coating before and after exposure to metal ions (see Figure 5) provides complementary information on the optical response of the immobilized chromophore because the optical path, scattering contribution, reference background, hydration state, and physical state of ARS differ between the dispersion and the coating. After drying on the glass substrate, the PU–ARS film displayed a broad visible signal in the spectral region where ARS absorbs. The profile differs from that of the liquid precursor because the measurement geometry, film hydration, scattering contribution, and reference background are different. Therefore, these solid-film spectra are used only as qualitative evidence of optical changes at the coating level, not as direct proof of ARS–metal complex formation.

After exposure to metal-enriched simulated seawater, the normalized solid-film spectra showed differences in profile and intensity. Hydration, salt deposition, surface morphology, refractive-index changes, and scattering may contribute to these variations. Accordingly, Figure 5 is used as a qualitative description of the coating-level optical response rather than to establish an ion-dependent molecular response or binding-affinity sequence.

Overall, the spectroscopic results support the conclusion that ARS retains its chromophoric character after incorporation into the polyurethane matrix and remains at least partially accessible to divalent metal ions under saline immersion conditions.

A direct comparison with aqueous ARS–metal complexes was not performed. Therefore, the solid-film spectra are not used to define the absorption spectra, colors, binding affinities, or coordination structures of discrete ARS–metal complexes in solution. Solution-phase titration and spectroscopic experiments under controlled pH and ionic-strength conditions would be required to separate intrinsic ARS–metal complexation from coating-level effects such as hydration, scattering, ion pairing, refractive-index variations, and matrix swelling.

**Figure 6:**
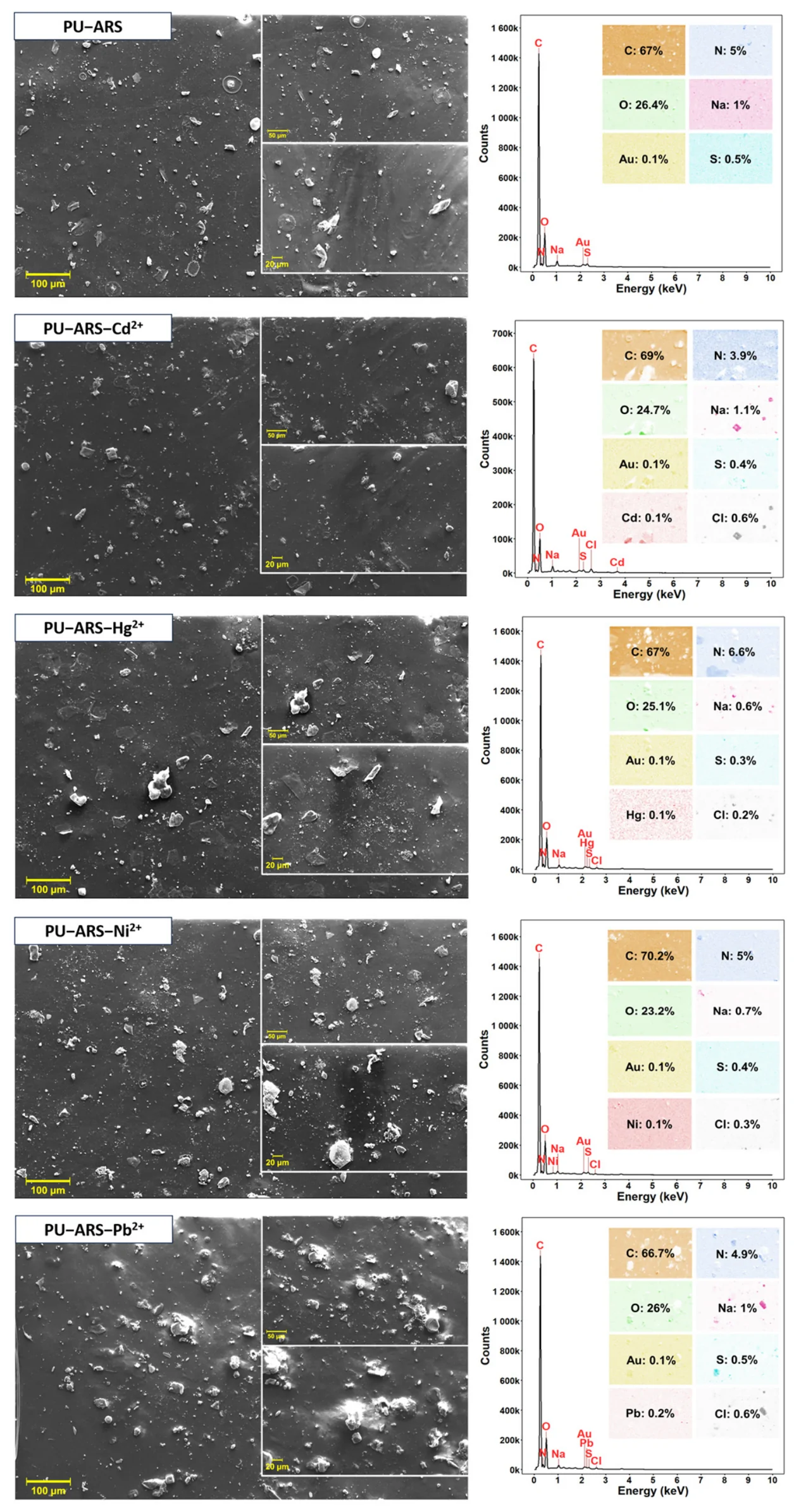
SEM micrographs and corresponding EDS spectra of PU–ARS coatings before and after 48 h exposure to simulated seawater containing Cd^2+^, Hg^2+^, Ni^2+^, or Pb^2+^ at 5 × 10^−4^ M. SEM images were acquired at 500×, 1000×, and 2000× magnification. EDS results are semi-quantitative and refer exclusively to the analyzed surface areas; Au arises from the sputter-coated conductive layer.

As a complementary surface characterization, SEM-EDS analysis was performed on PU–ARS coatings before and after exposure to metal-enriched simulated seawater (Figure 6).

The pristine coating showed a continuous morphology with dispersed particulate features and localized surface heterogeneities. After immersion in simulated seawater containing Cd^2+^, Hg^2+^, Ni^2+^, or Pb^2+^, the coatings retained a broadly comparable surface morphology, with no evident cracking, peeling, or large-scale delamination at the investigated length scale. The macroscopic retention of the coating on glass is plausibly related to substrate wetting, polar interactions with the glass surface, and cohesive film formation during room-temperature conditioning. This observation indicates film integrity under the selected immersion protocol, without implying quantified adhesion strength.

Furthermore, the present study did not include an independent determination of dry-film thickness or standardized peel/scratch testing before and after immersion. The stability assessment is therefore limited to macroscopic integrity, visual inspection, and retained colorimetric readability under the immersion protocol used here.

EDS spectra were dominated by C, O, and N, consistent with the organic PU–ARS matrix, while S, Na, and Cl were compatible with the sulfonated dye and residual salts from simulated seawater. Low-intensity Cd, Hg, Ni, and Pb signals were detected in the corresponding exposed coatings, supporting the qualitative presence of metal-containing species at the surface. These EDS signals should not be interpreted as quantitative evidence of metal uptake, binding capacity, or coordination stoichiometry; Au arises from the sputter-coated conductive layer. In addition, the quantification of light elements in EDS and the presence of the sputtered Au layer limit the use of these data to qualitative surface-level evidence. Collectively, ATR-FTIR and UV–visible spectroscopy support the presence of ARS-related spectral features in the PU coating, whereas SEM-EDS offers qualitative insights into surface morphology and the presence of metal-containing species after exposure. These data are consistent with coating-level perturbations induced by metal-containing solutions, but they do not quantify metal uptake or define the chemical state of the retained metals.

### 3.3. Colorimetric Response Under Single-Ion Exposure Conditions

The colorimetric response of PU–ARS coatings was evaluated after 48 h immersion in simulated seawater (ASTM D1141-98, pH 8.2) containing Pb^2+^, Cd^2+^, Ni^2+^, or Hg^2+^ at 5 × 10^−4^ M. A PU–ARS-coated slide immersed in metal-free simulated seawater under identical conditions was used as the reference. This design was intended to assess whether the immobilized ARS chromophore remained optically responsive within the PU matrix under saline conditions, and whether different divalent metal ions produced visually distinguishable color changes. The tested concentration should therefore be regarded as a proof-of-concept stress condition rather than as a target detection threshold.

**Figure 7:**
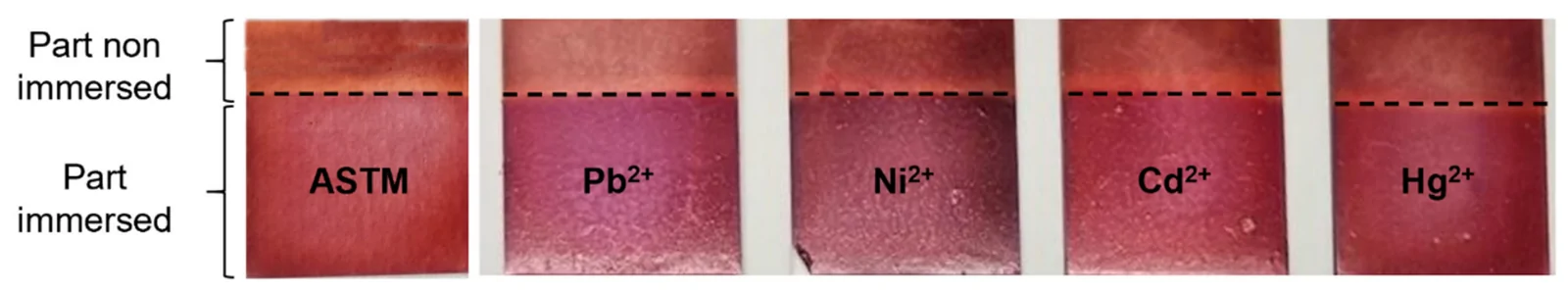
Photographic documentation of PU–ARS-coated glass slides after 48 h immersion in simulated seawater (ASTM D1141-98, pH 8.2) containing 5 × 10^−4^ M Pb^2+^, Ni^2+^, Cd^2+^, or Hg^2+^, compared with the metal-free seawater reference.

Accordingly, the ΔE values reported here describe the relative optical response under the selected single-ion conditions and should not be interpreted as evidence of analytical selectivity in multicomponent samples. Only endpoint measurements were obtained after a fixed 48 h exposure, and reversibility was not investigated. Therefore, the reported color represents the coating response accumulated over the selected exposure interval rather than an equilibrium measure of dissolved metal-ion concentration. Its magnitude may depend on exposure time, transport within the hydrated film, and the amount of accessible ARS. No concentration–response relationship can be derived from the present dataset. As shown in Figure 7, the reference coating retained its distinctive reddish hue following immersion in metal-free simulated seawater, thereby showing that the PU–ARS film remained optically discernible under the specified saline conditions. Exposure to metal-enriched seawater resulted in observable, ion-dependent color changes. Ni^2+^ and Cd^2+^ ions induced a significant darkening of the coated area, with exposed films exhibiting a transition toward more intense red-brown hues. Pb^2+^ exhibited a more pronounced pink-magenta displacement in comparison to the reference, whereas Hg^2+^ resulted in a less intense yet still discernible color variation. These visual distinctions suggest that the optical response of ARS molecules incorporated within the polyurethane matrix is affected by metal exposure. However, the observed response relates to an optical phenomenon occurring at the coating level, rather than serving as direct confirmation of a specific coordination geometry.

**Figure 8:**
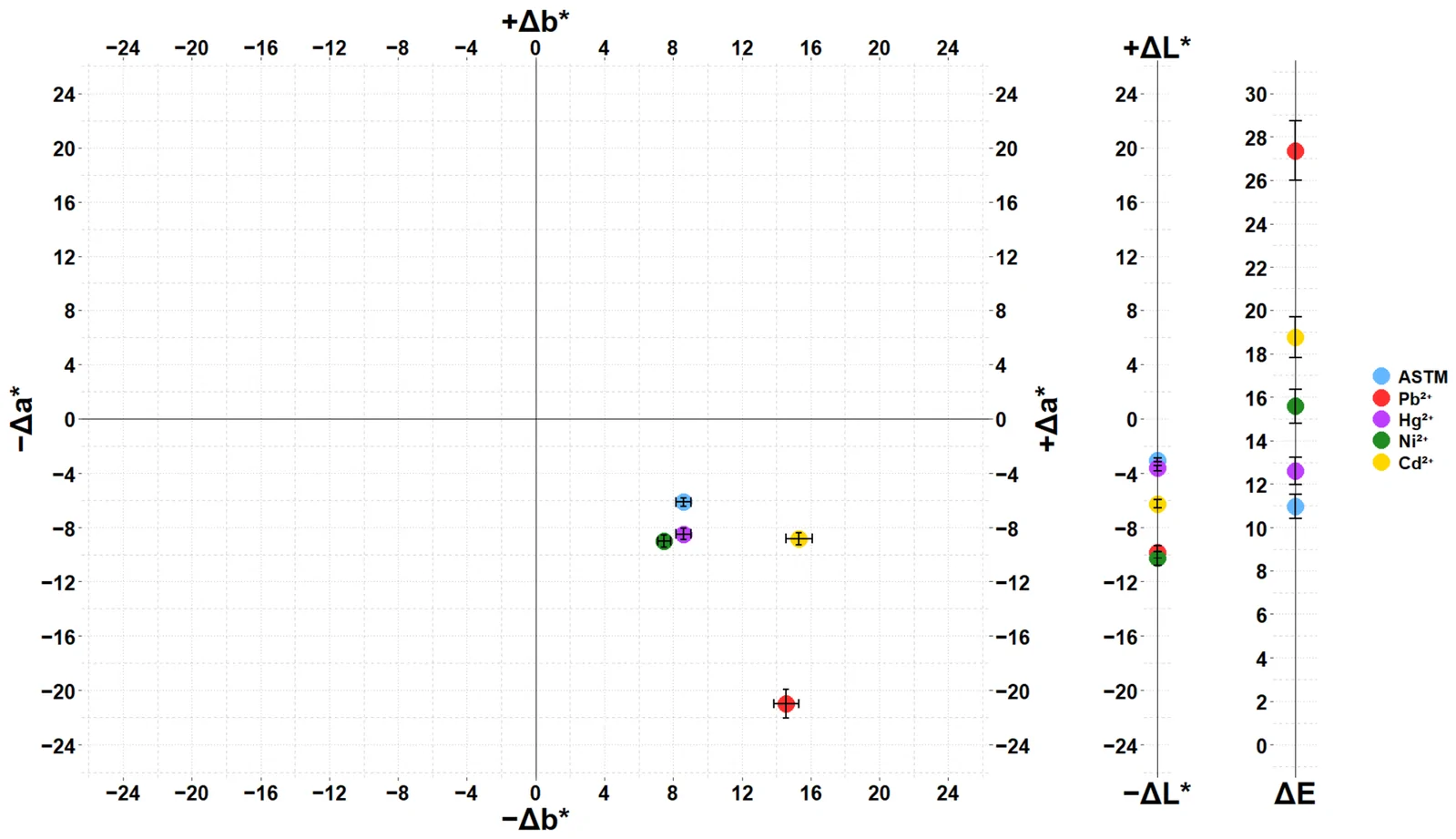
CIE L*a*b* color coordinates and total color difference (ΔE) for PU–ARS coatings after single-ion exposure to Pb^2+^, Cd^2+^, Ni^2+^, or Hg^2+^ in simulated seawater, relative to the metal-free seawater reference.

Instrumental color measurements within the CIE L*a*b* space corroborated the photographic observations (Figure 8, Table S1). All metal-exposed coatings exhibited negative ΔL* values in comparison to the metal-free seawater reference, indicating that exposure to divalent metal ions resulted in a reduction in lightness of the coating. This darkening effect was most pronounced for Ni^2+^ and Cd^2+^, consistent with their darker appearance in the photographs. Pb^2+^ produced the greatest overall chromatic displacement because its response involved combined changes in both lightness and chromatic coordinates, rather than a mere reduction in brightness alone. Among the four ions evaluated, Hg^2+^ exhibited the least pronounced color perturbation.

The total color difference adhered to the sequence Pb^2+^ > Cd^2+^ > Ni^2+^ > Hg^2+^ under the current single-ion conditions, with approximate ΔE values of 27, 19, 16, and 13, respectively. All values exceed the threshold associated with reliably perceptible color differences and support visual discrimination from the metal-free reference under controlled observation conditions. The ranking Pb^2+^ > Cd^2+^ > Ni^2+^ > Hg^2+^ refers only to the single-ion experiments carried out at 5 × 10^−4^ M. It should not be read as analytical selectivity. In a mixed-metal sample, different ARS–metal interactions, competition for accessible dye sites, ion pairing in the saline matrix, and matrix-mediated optical effects may produce non-additive color responses. Mixed-ion calibration was not performed in the present study and will be required before using the coating for sample classification beyond preliminary visual screening.

Because both the reference and metal-enriched media were prepared with ASTM D1141-98 substitute ocean water, major seawater ions such as Na^+^, Mg^2+^, Ca^2+^, and K^+^ were present throughout the experiments. This controls the bulk saline background but does not constitute an interference or mixed-metal selectivity study.

The comparison between UV–visible transmission and CIE L*a*b* reflectance data highlights the multi-parameter nature of the solid-film response. Solid-film UV–visible transmission and reflectance colorimetry probe different aspects of the coating response and need not vary in parallel, as the measured signal can be affected by absorption, scattering, surface reflectance, hydration, refractive-index changes, and lightness.

At this stage, the PU–ARS system should be considered a preliminary screening platform rather than a fully validated quantitative sensor. Analytical figures of merit, including calibration range, detection limit, quantification limit, response time, reversibility, and interference tolerance, cannot be established from experiments performed at a single metal concentration.

Because the response was evaluated after 48 h immersion, the current configuration is more appropriately described as a passive colorimetric screening interface than as a rapid-response sensor. Whether the coating can be adapted to short-term monitoring or is more appropriately deployed as an integrative exposure indicator requires time-resolved experiments that are beyond the scope of the current study. Nevertheless, the combination of visible color changes, measurable CIE L*a*b* shifts, and macroscopic coating readability after saline exposure supports the potential of PU–ARS films as simple colorimetric interfaces for preliminary heavy-metal screening in marine-like environments.

### 3.4. Saline Pre-Immersion and Retained Optical Responsiveness

The preservation of coating readability after saline exposure is relevant for a dye-containing coating, because continuous contact with aqueous saline media may promote swelling, dye redistribution or leaching, loss of optical contrast, and gradual deterioration of the polymer-substrate interface [42,43]. These effects are particularly important in colorimetric sensing materials, where the polymer matrix must simultaneously retain the chromophore and allow analyte transport. The response of polymer-based sensors is therefore strongly influenced by the polymeric phase, which acts both as a support for the sensing unit and as a medium governing analyte access [44]. Therefore, immobilization in PU is effective only if the matrix can balance two competing requirements: sufficient water access for ion transport and sufficient dye retention to preserve optical contrast.

After saline pre-immersion for 8, 16, 24, 48, and 72 h, the coatings retained visible responsiveness during the subsequent 48 h exposure to the selected metal-ion solutions. These observations indicate that an optically active fraction of ARS remained accessible after short-term contact with simulated seawater. The immersion media were not analyzed for released ARS or polymer, so dye leaching and coating mass loss were not quantified. The pre-immersion experiment therefore provides a short-term assessment of retained optical responsiveness rather than a measure of operational lifetime. Mass loss data, cyclic exposure experiments, and accelerated aging tests were not performed. Claims of complete dye retention, regeneration capacity, or long-term quantitative sensing performance would be premature based on the current data. Within these limits, the durability data support further evaluation of PU–ARS coatings as passive colorimetric interfaces for initial screening in saline environments. Glass slides were used here as a model support because they are transparent, chemically inert under the selected conditions, and compatible with transmission and reflectance measurements. Transfer of the coating to polymeric films, textiles, metallic supports, or marine-deployment substrates is possible only after substrate-specific assessment of wetting, adhesion, swelling, optical background, and probe retention.

## 4. Conclusions

Alizarin Red S-functionalized polyurethane coatings were prepared on glass substrates and evaluated as passive colorimetric interfaces for preliminary heavy-metal screening in simulated seawater. The hydrophilic PU matrix allowed the preparation of optically readable films. UV–visible spectroscopy and ATR-FTIR supported the presence of ARS-related optical and vibrational features in the PU–ARS system, whereas DLS described only the colloidal behavior of the precursor dispersions before film formation. The data are consistent with physical incorporation of ARS in the polymer matrix, but they do not define a unique binding structure.

Under single-ion exposure at 5 × 10^−4^ M in simulated seawater, Pb^2+^, Cd^2+^, Ni^2+^, and Hg^2+^ produced distinguishable color changes. Reflectance colorimetry gave approximate ΔE values of 27, 19, 16, and 13, respectively. This sequence describes the response under the specific single-ion conditions tested and should not be interpreted as analytical selectivity. The color response reflects coating-level effects involving ARS, the hydrated PU matrix, ion transport, reflectance, and lightness changes. After saline pre-immersion up to 72 h, the coatings remained macroscopically readable and retained optical responsiveness after subsequent exposure to metal-ion solutions. This supports short-term stability under the selected protocol, but it does not establish operational lifetime, reversibility, dye retention, or quantitative sensing performance. The coating should therefore be considered a proof-of-concept passive screening interface.

A practical format could be a disposable exposure coupon retrieved after a defined immersion period and read visually or by portable reflectance colorimetry, with positive responses subsequently verified by a reference analytical method. Calibration curves, detection limits, response kinetics, interference from coexisting ions, leaching analysis, and validation in real seawater are required before practical environmental use can be proposed.

## Data Availability

Data supporting the findings of this study are available from the corresponding authors upon reasonable request.

## Supplementary Materials

The following supporting information can be downloaded at: https://www.mdpi.com/article/10.3390/polym18182294/s1, Table S1. CIE L*a*b* coordinates and corresponding color differences of PU—ARS coatings before immersion and after exposure to metal-free or metal-containing simulated seawater.

## Abbreviations

The following abbreviations are used in this manuscript:

ARS	Alizarin Red S
PU	Polyurethane
