# Supplementary material for: Alizarin Red S-Functionalized Polyurethane Coatings as Passive Colorimetric Interfaces for Visual Heavy-Metal Screening in Simulated Seawater
Source: Polymers (Basel). 2026 Sep 19;18(18):2294. doi: 10.3390/polym18182294 (PMC13611583; doi:10.3390/polym18182294)
Supplement: Supplementary file 1 [file polymers-18-02294-s001.zip › polymers-4495621-supplementary.pdf]

Supplementary Materials

Alizarin Red S-Functionalized Polyurethane Coatings as Passive Colorimetric Interfaces for Visual Heavy-Metal Screening in Simulated Seawater

Silvia Sfameni [1,†], Mariam Hadhri [2,†], Giulia Rando [1], Agnese D'Agostino [2,3], Raphael Palucci Rosa [2], Giuseppe Rosace [2,3,‡], Valentina Trovato [2,3,*] and Maria Rosaria Plutino [1,*,‡]

[1] Institute for the Study of Nanostructured Materials – National Research Council (ISMN–CNR), URT Messina, c/o Department of ChiBioFarAm, University of Messina, Viale F. Stagno d'Alcontres 31, 98166 Messina, Italy;; silvia.sfameni@cnr.it (S.S.); giulia.rando@cnr.it (G.R.)

[2] Department of Engineering and Applied Sciences, University of Bergamo, Viale Marconi 5, 24044 Dalmine, Italy; mariam.hadhri@unibg.it (M.H.); agnese.dagostino@unibg.it (A.D.); raphael.rosa@unibg.it (R.P.R.); giuseppe.rosace@unibg.it (G.R.)

[3] Local CSGI (Inter-University Center for Colloid and Surface Science) Research Unit, 24044 Dalmine, Italy

* Correspondence: valentina.trovato@unibg.it (V.T.); mariarosaria.plutino@cnr.it (M.R.P.)

† These authors contributed equally to this work.

‡ These authors contributed equally to this work as senior authors.

Table S1. CIE L*a*b* coordinates and corresponding color differences of PU–ARS coatings before immersion and after exposure to metal-free or metal-containing simulated seawater.

Sample	L*	a*	b*	ΔL*	Δa*	Δb*	ΔE*
**PU–ARS Ref.**	53.95	31.42	12.77	0.000	0.000	0.000	0.000
**ASTM**	50.92	40.02	6.65	−3.03	+8.61	−6.12	**10.98**
**Pb^{2+}**	44.14	46.001	−8.20	−9.80	+14.58	−20.97	**27.36**
**Cd^{2+}**	47.69	46.74	3.96	−6.26	+15.32	−8.81	**18.75**
**Ni^{2+}**	43.67	38.90	3.77	−10.28	+7.48	−8.99	**15.58**
**Hg^{2+}**	50.32	40.03	4.29	−3.63	+8.61	−8.48	**12.61**

Note: PU–ARS Ref. denotes the PU–ARS-coated glass slide before immersion and was used the reference for calculating ΔL*, Δa*, Δb*, and ΔE*. **ASTM** denotes the PU-ARS-coated glass slide after immersion in metal-free ASTM D1141-98 simulated seawater at pH 8.2. **Pb^{2+}, Cd^{2+}, Ni^{2+}**, and **Hg^{2+}** denote PU–ARS-coated glass slides after immersion in ASTM D1141-98 simulated seawater at pH 8.2 containing the corresponding metal ion.
